# Manual versus automated blood sampling: impact of repeated blood sampling on stress parameters and behavior in male NMRI mice

**DOI:** 10.1177/0023677214541438

**Published:** 2014-10

**Authors:** A C Teilmann, Otto Kalliokoski, Dorte B Sørensen, Jann Hau, Klas S P Abelson

**Affiliations:** 1 Department of Experimental Medicine, Faculty of Health and Medical Sciences, University of Copenhagen, Copenhagen, Denmark; 2 Department of Veterinary Disease Biology, Faculty of Health and Medical Sciences, University of Copenhagen, Copenhagen, Denmark

**Keywords:** catheterization, corticosterone, anxiety, refinement, reduction

## Abstract

Facial vein (cheek blood) and caudal vein (tail blood) phlebotomy are two commonly used techniques for obtaining blood samples from laboratory mice, while automated blood sampling through a permanent catheter is a relatively new technique in mice. The present study compared physiological parameters, glucocorticoid dynamics as well as the behavior of mice sampled repeatedly for 24 h by cheek blood, tail blood or automated blood sampling from the carotid artery. Mice subjected to cheek blood sampling lost significantly more body weight, had elevated levels of plasma corticosterone, excreted more fecal corticosterone metabolites, and expressed more anxious behavior than did the mice of the other groups. Plasma corticosterone levels of mice subjected to tail blood sampling were also elevated, although less significantly. Mice subjected to automated blood sampling were less affected with regard to the parameters measured, and expressed less anxious behavior. We conclude that repeated blood sampling by automated blood sampling and from the tail vein is less stressful than cheek blood sampling. The choice between automated blood sampling and tail blood sampling should be based on the study requirements, the resources of the laboratory and skills of the staff.

Blood sampling from laboratory animals is a routine procedure in biomedical research. However, in the mouse, which is the laboratory animal used by far the most in the European Union,^1^ serial blood sampling is complicated by the limited blood volume of the animal as well as by animal welfare concerns and practical difficulties related to puncturing available venous access sites repeatedly. Refinement of routine blood sampling techniques in mice is thus important for improving animal welfare as well as the quality of the blood samples obtained.

Several methods for manual blood sampling in mice are available. Puncturing of the orbital venous plexus is controversial, and it is debated whether it can be considered an ethical method due to the risk of severely damaging the orbit and associated tissue.^2,3^ Furthermore, due to its potential severity this method should be performed under general anesthesia, which not only prolongs the blood sampling procedure, but certain anesthetic agents may also influence the metabolism and clearance of drugs and endogenous substances.^4,5^ This method may thus be inappropriate in toxicology and pharmacokinetic studies. The present study considered two other widely recognized methods for manual blood sampling: facial vein phlebotomy and caudal vein phlebotomy.

Facial vein phlebotomy is a popular blood sampling method in mice due to its simplicity and short duration. However, because of the considerable risks of inner ear penetration and laceration of facial muscles and nerves, a skilled phlebotomist is required in order to avoid post-procedural complications.^6^ Different methods for sampling blood from the caudal veins and artery of the tail, such as tail clip and tail incision^7–9^ or simply puncturing the vein/artery with a needle,^10^ are also frequently used in mice. Regardless of the sampling method, manual blood sampling will inevitably cause a stress response from the vascular puncture as well as from the handling and restraint of the animal,^11,12^ which may affect the experimental outcome.^13^

A relatively new technique for repeated blood sampling in mice is automated blood sampling (ABS) through a vascular catheter. The jugular vein^14–16^ and common carotid artery^17,18^ are commonly used for this purpose. After surgery, blood samples may be automatically obtained through the catheter without interfering with the animal at the time of sampling, hence making the blood sample unaffected by handling and restraint of the animal. This may increase animal welfare and reduce sampling-associated stress. ABS has been validated for use in rats, with negligible impact on stress-associated hormone levels,^19,20^ which is why this method is regarded as minimally stressful in this species. In mice, however, the stress response associated with ABS has at present not been fully investigated. Furthermore, this technique poses much greater technological challenges, when applied in smaller animals.

The aim of the present study was to investigate the physiological and behavioral responses to ABS in comparison with the responses to facial vein (cheek blood) and caudal vein (tail blood) phlebotomy. The hypothesis was that ABS is less stressful to the animal than is repeated manual blood sampling by cheek blood and tail blood.

## Animals, materials and methods

The experiments were approved by the Animal Experiments Inspectorate under the Danish Ministry of Food, Agriculture and Fisheries (license number: 2012/561-169). The animals were at all times handled by trained personnel in accordance with the *Guide for the Care and Use of Laboratory Animals*^21^ in a fully Association for Assessment and Accreditation of Laboratory Animal Care (AAALAC) International accredited facility.

In total, 60 male BomTac:NMRI mice, six to eight weeks old, weighing 39.6 ± 3.6 g (mean ± SD) were purchased from Taconic, Ry, Denmark. Based on the expected mean differences in concentrations of plasma corticosterone (from experience an increase of >40% was considered biologically relevant), a sample size of eight animals per group was considered appropriate, as estimated using a power equation^22^ on data from pilot studies with β = 0.20, α = 0.05, SD = 0.50. The mice were randomly divided into four groups: one control group (Group 1, *n* = 16), one group sampled by ABS (Group 2, *n* = 8), one group sampled by facial vein phlebotomy (Group 3, *n* = 18) and one group sampled by caudal vein phlebotomy (Group 4, *n* = 18). The control group was further split into two groups, such that eight mice served as controls for weight data, fecal corticosterone dynamics and behavior (Group 1a), and the other eight mice served to provide baseline plasma corticosterone levels from undisturbed mice (Group 1b). This was done to eliminate the potential influence on behavior (Group 1a) and plasma corticosterone levels (Group 1b) by previous sampling of other parameters. Except for these recordings, the control mice were left undisturbed.

Due to technical difficulties with the ABS device, blood from one mouse in Group 2 was not successfully collected. This mouse was thus eliminated from the study, resulting in a group size of seven mice. The manual sampling methods required twice as many mice (see Blood sampling section) and an additional two mice were added to both groups to secure adequate group sizes in case of sampling failure, resulting in 18 mice in Groups 3 and 4 (Table 1).

**Table 1.** Study outline.

	Day							
Group	–6	–5	–4	–3	–2	–1	0	1
1 a (control) *n* = 8				BW, DFI, DWI	BW, DFI, DWI	BW, DFI, DWI	BW, DFI, DWI Cage change	BW, DFI, DWI Fecal sampling Triple test
1 b (control) *n* = 8							Blood sampling	
2 (ABS) *n* = 7	BW, DFI, DWI	BW, DFI, DWI	BW, DFI, DWI	BW, DFI, DWI Catheterization	BW, DFI, DWI	BW, DFI, DWI	BW, DFI, DWI Cage change Blood sampling	BW, DFI, DWI Fecal sampling Triple test
3 (cheek) *n* = 18				BW, DFI, DWI	BW, DFI, DWI	BW, DFI, DWI	BW, DFI, DWI Cage change Blood sampling	BW, DFI, DWI Fecal sampling Triple test
4 (tail) *n* = 18				BW, DFI, DWI	BW, DFI, DWI	BW, DFI, DWI	BW, DFI, DWI Cage change Blood sampling	BW, DFI, DWI Fecal sampling Triple test

In total, 60 mice were randomly divided into four groups; one control group (control), one group sampled by automated blood sampling (ABS), one group sampled by facial vein phlebotomy (cheek) and one group sampled by caudal vein phlebotomy (tail). The control group was split into two groups in order to obtain behaviors in the triple test (1 a) and plasma corticosterone levels (1 b), not influenced by previous handling. The control mice were, except for the recording indicated in the table, otherwise left undisturbed. Body weights (BW), daily food intake (DFI) and daily water intake (DWI) were recorded each morning throughout the experiment from three days prior to (1) surgery at day –3 (Group 2), (2) blood sampling at day 0 (Groups 3 and 4) or (3) control behavioral analysis at day 1 (Group 1). The mice of Groups 2–4 were transferred to clean cages and the bedding was then collected after 24 h of blood sampling. The eight mice of Group 1 was transferred in the same way to clean cages in the morning one day prior to the behavioral analysis and the bedding was collected after 24 h of no intervention.

In relation to the experimental procedures, the mice were handled by cupping, as this has been shown to reduce handling-associated anxiety in laboratory mice.^23^

### Housing

Upon arrival at the facility, the mice were singly-housed and allowed to acclimatize for two weeks before experimentation. Aspen chips (Tapvei Oy, Kortteinen, Finland) were used as bedding material. Bite bricks (Tapvet®, Kortteinen, Finland), nesting materials (Lillico, Horley, UK) and cardboard houses (Brogaarden, Gentofte, Denmark) were used for environmental enrichment. Feed (Altromin 1314; Altromin GmbH & Co KG, Lage, Germany) and acidified tap water were provided ad libitum, and a diurnal rhythm was maintained with a 12:12 h light–dark cycle, with artificial light from 06:00 h and 30 min of twilight before lights were turned off or on. Cage temperature was kept at 22℃, relative humidity at 55%, and the air was exchanged 75 air changes per hour.

### Catheterization

The mice in Group 2 were catheterized in the right common carotid artery. Anesthesia was induced with 5% isoflurane (Forene®; Abbott Scandinavia, Stockholm, Sweden) conveyed in 100% oxygen, and maintained at 2.5–3.0% isoflurane in 100% oxygen. Mice were provided anesthesia by spontaneous breathing through an anesthetic face mask (AgnTho’s AB, Lidingö, Sweden). An approximately 5 mm long midline incision was made in the ventral neck region, and the fascia and mandibular salivary glands were carefully separated in the midline by blunt dissection. The carotid artery was approached and carefully dissected. Two 6–0 vicryl sutures (Ethicon, St-Stevens-Woluwe, Belgium) were placed around the vessel, one rostral suture just beneath the bifurcation and the other caudally. The rostral suture was completely tied. On the caudal suture, the first half of a surgical knot was loosely tied but not tightened. A small hole was cut in the artery and an arterial catheter (SAI Infusion Technologies, Lake Villa, IL, USA) was inserted in the artery. The caudal suture was tied around both catheter and vessel without impeding catheter patency. The rostral and caudal sutures were cross-tied and the catheter was checked for patency and flushed with 50 µL heparinized saline (25 IU/mL). Finally, the catheter was tunneled subcutaneously to the mid scapular region of the neck, and the skin was sutured in two layers. Surgery was completed before noon and performed under aseptic conditions.

The mice were given pre-emptive analgesia consisting of 1 mg/kg body weight (BW) buprenorphine (Temgesic; Schering-Plough Europe, Brussels, Belgium) mixed in a commercially available nut paste (Nutella®; Ferrero, Pino Torinese, Italy), given for voluntary ingestion one hour prior to surgery and then once daily for two days post-surgery. Buprenorphine tablets (0.2 mg) were crushed to a fine powder before mixing with 1 g nut paste to ensure an even concentration of 0.2 mg buprenorphine/g nut paste. The dose was based on published recommendations,^24,25^ as well as previous experiences.^26,27^ The mice were offered pure nut paste once daily for three days prior to surgery to habituate them to the flavor, as this has been shown to promote voluntary ingestion.^26,28^ Furthermore, to ensure optimal analgesia while recovering from surgery, the mice were given 0.1 mg buprenorphine/kg BW subcutaneously during anesthesia at the end of surgery.

A silicone harness (Instech Laboratories Inc, Plymouth Meeting, PA, USA) was put on the mice after surgery, through which the catheter was guided and further connected to the ABS device (AccuSampler®; VeruTech AB, Lund, Sweden). The catheter was flushed automatically, every 30 min, throughout the experiment with 65 µL of 25 IU/mL heparinized saline to maintain patency.

### Blood sampling

The eight mice in Group 1b were concussed and decapitated for blood sampling. Four mice were sacrificed at 09:00 h and four at 18:00 h. Trunk blood was collected within 30 s of concussion. The blood samples from these mice were used to obtain baseline levels of plasma corticosterone from non-stressed mice.

The mice in Group 2 were allowed to recover for three days after surgery before initiating blood sampling. This was done to completely remove the influence of surgical stress on the blood samples and to habituate the mice to the ABS system before blood sampling, as a previous study has shown that three days recovery after arterial catheterization is necessary for corticosterone levels, daily food consumption and BW to return to pre-surgical levels.^18^ During this period, the mice were frequently examined to ensure proper recovery. Three days post-surgery, starting at 09:00 h, the mice were blood sampled every third hour over 24 h. Eight blood samples of 25 µL were collected automatically in microtubes (VeruTech AB). An additional waste volume of 5 µL was lost with each blood sampling, resulting in a total blood loss of approximately 240 µL per mouse. Based on the BW, the total blood volumes could be estimated to 2.5–3.0 mL. Thus, a total blood loss of 240 µL corresponds to 7.9–9.5% of the total blood volume. After each blood sample, 25 µL of 25 IU/mL heparinized saline was infused automatically to replace the lost blood. The blood sampling was computer-controlled and no personnel were present in the room during sampling in order to avoid influence on the circulating levels of corticosterone in the samples from external stressors. After each blood sample had been obtained, the mice were visually inspected to ensure that the procedure had run properly.

The mice in Groups 3 and 4 were sampled for blood manually by facial and tail vein phlebotomy, respectively. Since eight blood samples from the same animal would have constituted too large a blood loss, twice as many mice were utilized in Groups 3 and 4. Each mouse was sampled four times, twice in the day time and twice during the night. The mice were randomized within groups with regard to sampling occasion, and distributed so that each time point was represented by eight mice. The mice in Group 3 were briefly restrained by scruffing of the neck skin, and blood was collected from the cheek using a Goldenrod lancet (MEDIpoint Inc, Mineola, NY, USA). Consecutive blood samples were collected, alternating between left and right sides, and care was taken to puncture the facial vein and not the superficial temporal vein. After blood collection, the stasis (restraint) was released to stop the bleeding.

The mice in Group 4 were restrained using a homemade tail vein fixation platform, where the mouse was put on a box and the tail was let through a V-shaped opening in a plastic pane. Only the tail was handled during the venipuncture. The lateral caudal vein was punctured distally on the middle third part with a 25 gauge needle. The vein was gently stroked in a proximal to distal direction in order to massage the blood out. The blood was collected alternately from the left and right lateral caudal veins. The bleeding stopped instantly when the tail was no longer stroked.

Samples of approximately 50 µL were collected in heparin coated tubes (BD Microtainer; BD Inc, Franklin Lakes, NJ, USA), and the total blood loss was estimated to be 200–250 µL per mouse (Groups 3 and 4). The total blood loss thus maximally constituted 9.9% of the total blood volume.

The blood samples were centrifuged at 10,000 × g for 10 min to isolate plasma, which was then stored at −21℃ until analysis. Plasma corticosterone levels were quantified with an enzyme-linked immunosorbent assay (ELISA) (EIA-4164; DRG Diagnostics, Marburg, Germany) in accordance with the manufacturer’s instructions.

### Fecal sampling and weight recording

Before blood sampling, the mice in Groups 2–4 were transferred to clean cages and after 24 h of blood sampling the bedding was collected. Similarly, the eight mice in Group 1a were transferred to clean cages in the morning one day prior to the behavioral analysis and the bedding was collected after 24 h of no intervention. All fecal pellets were separated from the bedding and stored at –21℃ until extraction. The total excreted levels of fecal corticosterone metabolites (FCM) were extracted and quantified by ELISA (EIA-4164; DRG Diagnostics), as described previously.^17^

BW, daily food intake (DFI) and daily water intake (DWI) were recorded each morning throughout the experiment from three days prior to surgery (Group 2), manual blood sampling (Groups 3 and 4) or control behavioral analysis (Group 1a).

### Behavioral recording

After 24 h of blood sampling (Groups 2–4), the mice were subjected to a previously validated behavioral test, referred to as the triple test,^29^ for 15 min. The control mice (Group 1a) were subjected to the triple test without previous blood sampling. The triple test consisted of an open field (OF), an elevated plus maze (EPM) and a light–dark box (LDB) combined into one maze, elevated 45 cm from the floor and designed as described elsewhere.^29^ The OF was a 24.0 × 47.5 × 47.5 cm (h × w × d) light grey arena with white walls and black lines that divided the OF into outer zones (OZ) and inner zones (IZ). From the OF the maze continued to the EPM, which consisted of four 21 cm long dark grey arms, two closed arms (CA) and two open arms (OA). The heights of the CA walls were 30 cm and the center of the EPM measured 8.5 × 8.5 cm. The LDB measured 22 × 21 × 14.5 cm in the dark compartment (LDB-D), which was painted black and covered with a lid, and 22 × 21 × 23 cm in the light compartment (LDB-L), which was painted white and brightly illuminated by a lamp (50 W) placed directly above the chamber. Openings between compartments were 7 × 7 cm. The room was dimly illuminated so that the artificial light provided 8.0–36.0lx inside the OF, 8.0–42.4lx in the EPM and 5.0–20.3lx in the LDB-D. The bright illumination of the LDB-L provided 1276–2590lx. Illuminance was measured at the floor level in the respective compartments.

A mouse was placed centrally in the OF and allowed to explore the maze for 15 min. Two digital cameras (GZ MG6800; JVC, Yokohama, Japan) were placed on tripods (JVC), one covering the OF and EPM and one covering the LDB. The maze was cleaned with a cleaning solution (Neutral detergent; Unilever Danmark A/S, Copenhagen, Denmark) and paper towels between trials. No mice fell off the OA of the EPM.

Behaviors were scored manually using a computer program for the transcription and timing of behavioral observations (Etholog 2.2; Eduardo B Ottoni São Paulo, SP, Brazil). Behaviors were recorded as either state events (SE) or instant events (IE), where SE were defined as behaviors with a duration, and IE were defined as events with times of occurrence.^30^

If a mouse placed its four paws into a compartment this was counted as an entry. Time spent in the compartments OF IZ, OF OZ, EPM CA, EPM OA, LDB-L and LDB-D were timed as SE, and the number of entries into the compartments OF IZ, OF OZ, EPM CA, EPM OA, LDB-L and LDB-D were counted as IE. The time spent in the central square of the EPM was almost non-existent and was, therefore, merged with the time spent in the EPM CA. Risk assessments of the EPM OA and LDB-L were defined as IE, and were counted if only the front paws were placed in either the EPM OA or the LDB-L before returning to the EPM CA or LDB-D.

After the behavioral analysis, the mice were euthanized with 5% isoflurane.

### Statistics

Data were analyzed using SPSS Statistics 20 (IBM, Armonk, NY, USA) and tested for normality using Shapiro-Wilk’s test on either non-transformed or log-transformed data. Normally distributed data-sets were analyzed with either a one-way analysis of variance (ANOVA) or a two-way ANOVA for comparing the overall difference within and between groups, and with Tukey’s post hoc tests. Levene’s test of equality of sample variances was conducted to test whether the variances were equal across groups. Statistics are presented as an F value, F(df) or F(df_w_, df_b_), where df are the degrees of freedom in a one-way ANOVA or the degrees of freedom within and between groups, respectively, in a two-way ANOVA. Weight data (BW, DFI, DWI) were subjected to a repeated measures ANOVA, with a Greenhouse-Geisser correction where appropriate.

Data-sets that did not follow a Gaussian distribution were analyzed with a Kruskal-Wallis test. Statistics are presented as a chi-square value, χ^2^(df), and an asymptotic *P* value.

Furthermore, a principal component analysis (PCA) was performed on behavioral data to investigate latent trends that could explain a possible association between variables. A Varimax rotation with Kaiser normalization was employed in order to facilitate interpretability of the underlying components. The optimal number of extracted components was determined from a Scree plot. The differences in how the groups expressed these components were evaluated using a multivariate ANOVA with Tukey’s post hoc tests. *P* values <0.05 were considered significant.

## Results

### Body weight and daily food and water intake

**Figure 1.**
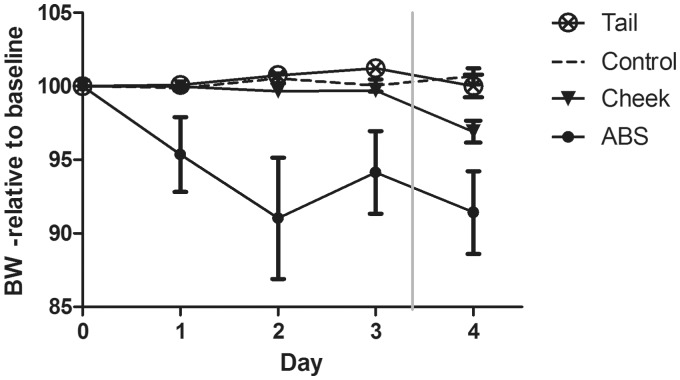
Body weight (BW) in relation to baseline values. Data are presented as percentages of the average weight recorded for three days prior to intervention. Sample points represent mean ± SEM for control mice (control), mice sampled by facial vein (cheek) and caudal vein (tail) phlebotomy and mice sampled automatically (ABS). The mice in the ABS group was catheterized at day 0 and allowed to recover until day 3, where blood sampling was initiated (vertical grey line).

The BW (Figure 1) changed significantly between groups (F(3,46) = 21.742, *P* < 0.001), where mice sampled by ABS lost weight compared with control mice (*P* < 0.001), mice sampled by facial vein phlebotomy (*P* < 0.001), and mice sampled by caudal vein phlebotomy (*P* < 0.001). Furthermore, a significant decrease in BW from days 3 to 4 was found for mice sampled by facial vein phlebotomy (F(1,17) = 17.378, *P* = 0.001).

**Figure 2.**
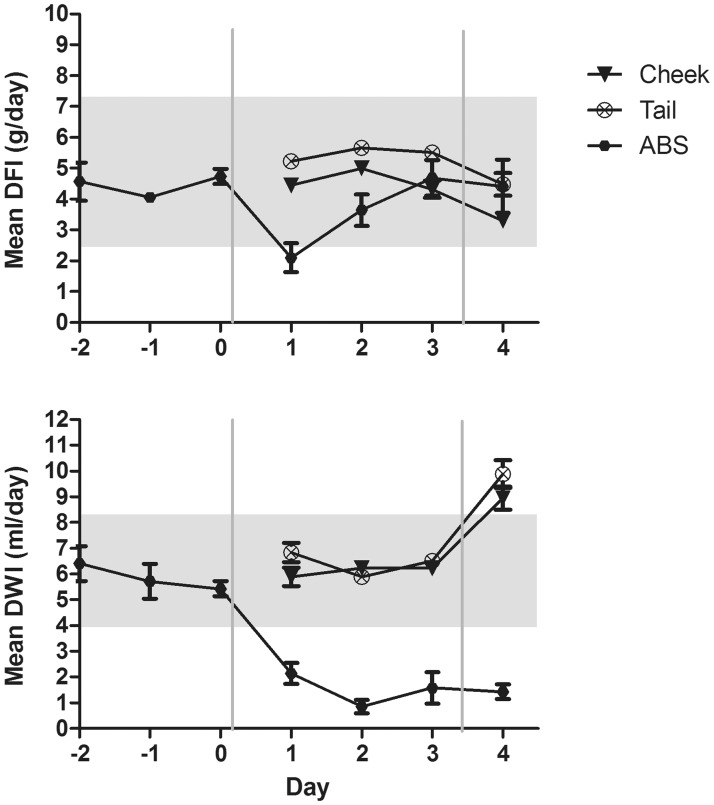
Daily food intake (DFI) and daily water intake (DWI), as shown from two days prior to surgery for mice sampled automatically (ABS), or two days prior to blood sampling for mice sampled by facial vein (cheek) and caudal vein (tail) phlebotomy. Surgery at day 0 and blood sampling at day 3 are indicated by grey lines. Data are presented as mean ± SEM. The grey areas represent 95% CI of DFI (2.53–7.41 g/day) and DWI (4.05–8.37 mL/day) of the control mice.

Overall, the DFI (Figure 2) differed significantly between groups (F(3,47) = 6.815, *P* = 0.001), where mice sampled by caudal vein phlebotomy on average consumed more feed than did mice sampled by facial vein phlebotomy (*P* = 0.001) and ABS (*P* = 0.026), although this did not differ significantly from the DFI of control mice. The DFI within the ABS group increased significantly from day 1 to day 3 (*P* = 0.046).

An overall significant difference in DWI (Figure 2) between groups was found (F(3,47) = 87.971, *P* < 0.001), where mice sampled automatically drank less than control mice (*P* < 0.001), mice sampled by facial vein phlebotomy (*P* < 0.001), and mice sampled by caudal vein phlebotomy (*P* < 0.001). Furthermore, a significant decrease in DWI was found for ABS mice from day –1 to day 2 (*P* = 0.023), day 3 (*P* = 0.035) and day 4 (*P = *0.016) and between day 0 and day 1 (*P* = 0.016), day 2 (*P* < 0.001) and day 4 (*P* = 0.002). The DWI of tail-bled mice was significantly higher than that of control mice from day 3 to day 4, and increased significantly within the group (*P* < 0.001) from days 3 to 4. The DWI of mice sampled by facial vein phlebotomy was not significantly different from that of control mice. However, it increased significantly within the group from days 3 to 4 (*P* < 0.001).

### Plasma corticosterone

**Figure 3.**
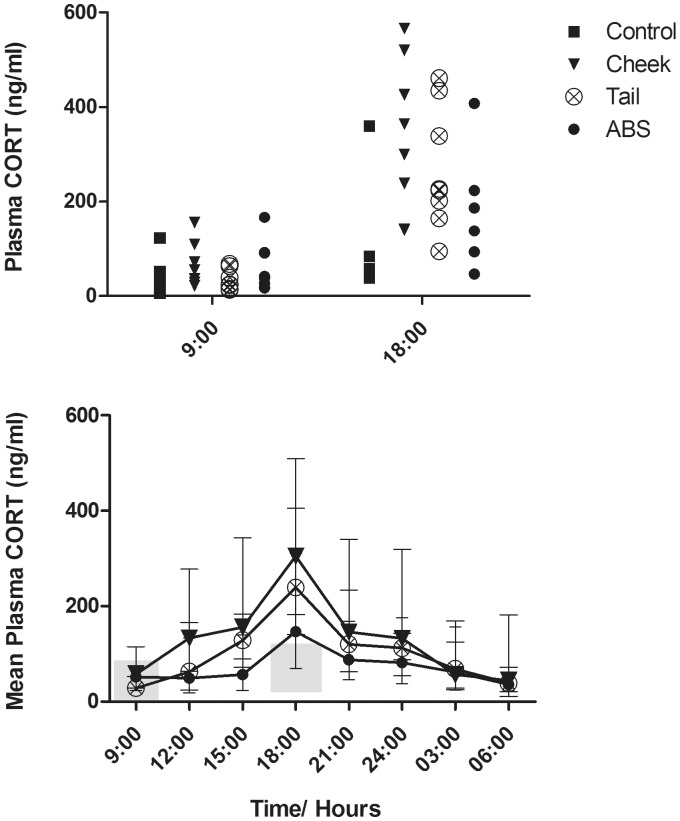
Plasma corticosterone levels during 24 h of blood sampling in mice sampled by facial vein (cheek) and caudal vein (tail) phlebotomy and automatically (ABS) and from control mice (control). Top: Actual values at 09:00 and 18:00 h, where control mice were sampled. Bottom: data are presented as mean ± SD. The grey areas represent 95% CI of the control mice at 09:00 h (2.89–91.51 ng/mL) and 18:00 h (30.42–118.27 ng/mL), respectively.

Log-transformed plasma corticosterone levels (Figure 3) were significantly different between groups (F(6,44) = 4.934, *P* = 0.001) at two time points. At 09:00 h, the plasma corticosterone concentrations of mice sampled by facial vein phlebotomy were significantly higher than those of control mice (*P* = 0.039). At 18:00 h, the plasma corticosterone concentrations of control mice were significantly lower than those of mice sampled by facial vein phlebotomy (*P* < 0.001) and tail vein phlebotomy (*P* = 0.001).

### Fecal corticosteroid metabolites

**Figure 4.**
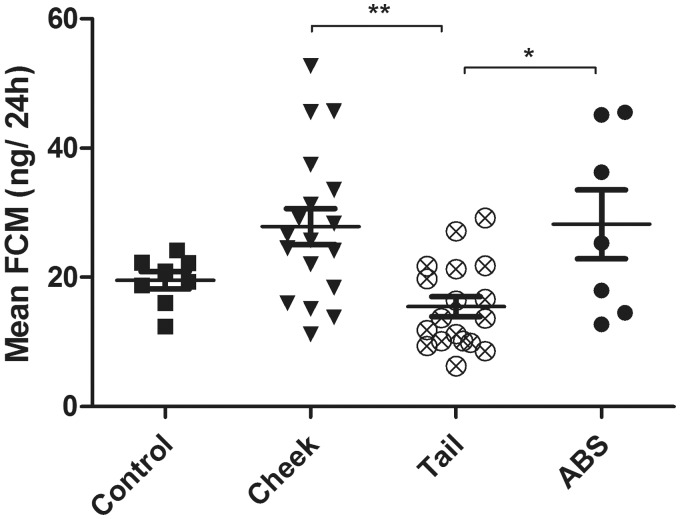
Fecal corticosterone metabolite (FCM) levels after 24 h of blood sampling in mice sampled by facial vein phlebotomy (cheek), caudal vein phlebotomy (tail) and automatically (ABS), and in control mice (control) after 24 h of no intervention. Data are presented as mean ± SEM. *P* values < 0.05 were considered significant (*) and *P* < 0.01 strongly significant (**).

The total amount of voided feces (mean ± SD) was 1.24 ± 0.21 g for control mice (Group 1 a), 1.07 ± 0.36 g for ABS mice, 1.08 ± 0.21 g for cheek-bled mice and 1.12 ± 0.21 g for tail-bled mice. FCM levels (Figure 4) were significantly different between groups (F(3) = 6.100, *P* = 0.001), where mice sampled by caudal vein phlebotomy excreted significantly less FCM compared with mice sampled by facial vein phlebotomy (*P* = 0.002) and ABS (*P* = 0.024).

### Behavior

**Figure 5.**
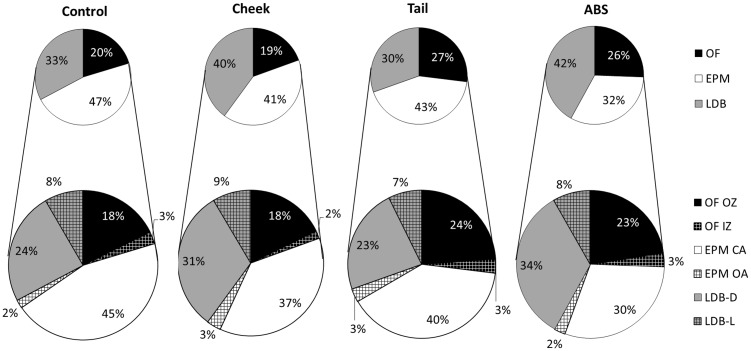
Time spent in the respective compartments of the triple test. The behavior of control mice (control), mice sampled by facial vein (cheek) and caudal vein (tail) phlebotomy and automatically (ABS) were studied in the triple test. The overall time spent in the open field (OF), elevated plus maze (EPM) and light–dark box (LDB) as well as the time spent further subdivided into open-field inner (OF IZ) and outer (OF OZ) zones, elevated plus maze open (EPM OA) and closed (EPM CA) arms, as well as in the light (LDB-L) and dark compartments (LDB-D) of the light–dark box. Data are presented as percentages of the total time spent (15 min). Mice sampled by cheek blood spent significantly less time than mice sampled by tail blood in the OF OZ (*P* = 0.047), and mice sampled by ABS spent significantly less time in the EPM CA than the control mice (*P* = 0.014).

The was a significant difference between groups regarding the total time spent (Figure 5) in the OF, (F(3) = 3.544, *P* = 0.023), EPM (F(3) = 3.537, *P* = 0.023) and LDB (F(3) = 4.134, *P* = 0.012), where mice sampled by facial vein phlebotomy spent significantly less time in the OF than mice sampled by caudal vein phlebotomy (*P* = 0.026). Mice sampled by ABS spent significantly less time in the EPM compared with control mice (*P* = 0.017), and mice sampled by caudal vein phlebotomy spent significantly less time in the LDB than mice sampled by facial vein phlebotomy (*P* = 0.032) and ABS (*P* = 0.039).

The division of time spent between the respective compartments was significantly different between the groups (F(18,114) = 2.093, *P* = 0.010), where mice sampled by facial vein phlebotomy spent significantly less time than mice sampled by caudal vein phlebotomy in the OF OZ (*P* = 0.047), and mice sampled by ABS spent significantly less time than control animals in the EPM CA (*P* = 0.014). When merging the aversive zones (OF IZ, EPM OA, LDB-L), no significant differences between groups appeared (F(3) = 0.023, *P* = 0.995). Similarly, when merging the safe zones (OF OZ, EPM CA, LDB-D), no significant differences between groups were found (F(3) = 0.024, *P* = 0.995).

The total number of entries, into all compartments, were not significantly different between groups (χ^2^(3) = 5.590, *P* = 0.133). However, the number of entries into the respective compartments differed significantly between groups with respect to the OF IZ (χ^2^(3) = 13.202, *P* = 0.004) and OF OZ (χ^2^(3) = 10.834, *P* = 0.013), where mice sampled by facial vein phlebotomy entered the OF IZ (mean ranks: ABS [31.21] > tail [28.41] > control [22.57] > cheek [13.60]) and OF OZ (mean ranks: tail [29.06] > ABS [27.29] > control [23.57] > cheek [14.27]) fewer times than the other groups.

The number of risk assessments in the EPM were significantly different between groups (χ^2^(3) = 10.337, *P* = 0.016), where mice sampled by ABS risk assessed less than the other groups (mean ranks: control [30.79] > cheek [25.03] > tail [23.59] > ABS [9.50]). There was no significant difference between groups in the number of risk assessments in the LDB (χ^2^(3) = 0.577, *P* = 0.902).

The PCA, revealed three components (Table 2) that cumulatively explained 71% of the total variance in the data-set. The components could be described as ‘exploration and curiosity’ (PC1), ‘transit behavior after risk assessment’ (PC2) and ‘anxiety’ (PC3). The expression of these components differed significantly between groups (F(9,123) = 3.477, *P* = 0.001), where mice sampled by facial vein phlebotomy expressed PC1 less than mice sampled by caudal vein phlebotomy (*P* = 0.014), and mice sampled by ABS expressed PC3 less than control mice (*P* = 0.001), mice sampled by caudal vein phlebotomy (*P* = 0.007) and marginally less than mice sampled by facial vein phlebotomy (*P* = 0.053). The duration of the behaviors as explained by PC2 did not differ significantly between groups.

**Table 2.** Factor loadings for three extracted components in a principal component analysis.

	Component		
	1	2	3
OF OZ	0.853		
OF IZ	0.788		
EPM CA			0.897
EPM OA	0.474		
LDB-D	−0.665		−0.671
LDB-L		0.746	
Risk ass EPM		0.742	
Risk ass LDB		−0.718	
Total NE	0.652	0.597	

Components were extracted using a Varimax rotation with a Kaiser normalization. Coefficients below 0.450 are not shown. OF OZ: open field outer zone, OF IZ: open field inner zone, EPM CA: elevated plus maze closed arms, EPM OA: elevated plus maze open arms, LDB-D: light–dark box dark compartment, LDB-L: light–dark box light compartment, Risk ass EPM: risk assessments in the elevated plus maze. Risk ass LDB: risk assessments in the light–dark box, Total NE: total number of entries.

## Discussion

In mice, the facial and caudal veins are commonly used for blood sampling, and surgical cannulation for ABS is becoming increasingly popular for serial blood sampling in rodents.^14,15,19,31,32^ Some studies have evaluated the impact of different blood sampling techniques on animal welfare^6,7,33,34^ and reliability of results.^35,36^ However, this is the first comparison of traditional manual blood sampling methods with the more recently developed ABS technique in mice. Furthermore, this study assessed animal welfare after repeated blood sampling, which is essential as serial blood sampling may have a serious impact on animal welfare.^2,37^

The present study demonstrated that the BW and DFI of mice sampled by ABS decreased as a consequence of surgery. Surgery is a potent stressor,^38^ and post-surgical BW reduction is common in mice.^17,39,40^ Catheter implantation is generally classified as a procedure of moderate severity^41^ with a mild pain potential.^42^ The analgesic regimen, used in the present study, is considered adequate for mild to moderate pain^42^ and has previously been validated to be appropriate for surgical procedures in rats and mice.^17,26,43,44^ However, numerous factors including the degree of tissue trauma, proficiency of the surgeon, surgery duration and degree of aseptic technique may influence the physiological response to the surgery. In the present study, carotid catheterization was performed under aseptic conditions by a skilled surgeon. A previous study in our laboratory showed that three days’ recovery is necessary after carotid catheterization in mice for BW and DFI to return to normal levels.^18^ The DFI of mice in the ABS group increased to pre-surgical levels on day 3 in the present study. However the BW of the ABS mice did not return to baseline levels within the studied period of time, possibly because of the initiation of blood sampling on day 3 post-surgery. Blood sampling elicited a small non-significant decrease in BW and DFI in ABS mice. A significant decrease in BW in relation to blood sampling was, however, evident in mice sampled by facial vein phlebotomy. A small non-significant decrease in BW from days 3 to 4 was also evident in mice sampled by caudal vein phlebotomy. The blood sampling may, thus, have an impact on the BWs of mice, when blood is sampled repeatedly from the cheek and to a lesser extent when it is sampled automatically or from the tail.

Mice sampled by tail blood consumed more feed on average when compared with cheek-bled and ABS mice, however, this was not significantly different from the DFI of control mice. Tail-bled mice were generally consuming more feed prior to blood sampling as well, which was why we considered this finding to be unrelated to the blood sampling.

The DWI decreased significantly after surgery in the ABS mice and remained at a significantly lower level compared with pre-surgical levels and the DWI of the other groups. This may be explained by the saline infused by the automated blood sampler to maintain catheter patency, resulting in reduced thirst and drinking behavior, as the infused fluid (65 µL every 30 min) necessitates less oral fluid intake. Careful monitoring of the animals in such an automated set-up is crucial in order to avoid fluid retention, which may lead to kidney failure or pulmonary edema. The infusion volume used in the present study was based on estimations of the body’s capacity to absorb fluid;^2^ and no complications resulting from the volume infused were observed in any of the mice.

The manually sampled mice exhibited increased water consumption during the day of blood sampling, although the DWI of only the tail-bled mice was significantly different from that of the control animals. The increased water consumption may have compensated for the loss of fluid caused by the blood samplings. However, frequent handling of the cages and thereby also of the water bottles, when collecting the mice for blood sampling, may have resulted in dripping from the water bottles, contributing to the measured increase in DWI.

When exposed to a stressor, the body responds by activation of the hypothalamic–pituitary–adrenal axis and through the release of glucocorticoids into the circulation.^12,45^ In mice the biologically-active glucocorticoid is corticosterone,^46,47^ which may be quantified in blood samples or as corticosteroid metabolites in feces.^48–50^ In the present study, mice subjected to manual blood sampling had significantly higher plasma corticosterone levels than did the control mice. Normally, in the absence of major stressors, plasma corticosterone will express a clear diurnal rhythm that peaks around dusk at approximately 200 ng/mL and then gradually declines during the night to levels below 50 ng/mL, which will then start to re-increase during the early afternoon.^47,51^ In the present study, a diurnal rhythm was seen in all groups. Plasma corticosterone levels from control mice and mice subjected to ABS ranged within normal levels,^51^ but mice sampled by facial vein phlebotomy had significantly elevated levels of plasma corticosterone at 09:00 and 18:00 h compared with the control mice. Mice subjected to tail vein phlebotomy had elevated plasma corticosterone levels at 18.00 h. Although the mice sampled manually for blood expressed significantly elevated concentrations of corticosterone in the circulation, their circadian rhythm was not disrupted.

Unlike plasma corticosterone, which may vary from moment to moment in circadian rhythms, the FCM levels, as measured in the present study, reflect the cumulative excretion of glucocorticoid metabolites over 24 h. This method may attenuate peak secretions,^20,52^ but sampling single pellets at certain time points may be unreliable due to high variation and individual differences in glucocorticoid concentrations in fecal pellets.^53,54^

The lag time from a peak in blood to the subsequent excretion in feces is approximately 9 h in mice;^48,50^ which is why the FCM levels in the mice subjected to ABS reflected not only the effects of the blood samplings but also of the last 9 h of recovery. This may have contributed to the elevated levels compared with the FCM levels of the tail-bled mice, as plasma corticosterone was not significantly increased during ABS. The increase in FCM levels in the cheek blood group, compared to the levels of the tail bled mice, is likely a reflection of the time of blood sampling and consequently the sampling method alone. Cheek blood sampling appears, thus, to result in higher stress levels than does tail blood sampling. However, the FCM levels of the control mice were not significantly different from those of the experimental groups. Although FCM quantification is well-studied and generally acknowledged as a valid biomarker of stress in rodents,^48,49^ some studies have shown that it is less sensitive in detecting the effect of minor stressors in mice.^17,20,39^ It is possible that the blood sampling in the present study resulted in minor stress levels which could not be detected by FCM quantification.

The OF, EPM and LDB are three well recognized and validated tests for assessing anxiety-related behaviors in mice and rats.^55–58^ However, it has been suggested that these tests are difficult to compare, as they measure different aspects of anxiety.^59^ Furthermore, intra-individual variation makes comparison between behavioral tests unreliable, and previous exposure to one test may affect the animal’s behavior in successive tests, usually by inhibiting actions.^59–61^ The triple test combines the OF, EPM and LDB in one test, hence increasing reliability by reducing variation between test batteries, and by minimizing potentially confounding effects of repeated testing.^62^ Although the triple test is a rather new test paradigm, it has been pharmacologically validated^59,60^ and has proven useful in assessing mouse phenotypes.^29^ In the present study, it was used to identify enhanced anxiety-related behaviors as an effect of the different repeated blood sampling techniques.

No overall difference between groups in their preferences of merged safe zones (OF OZ, EPM CA, LDB-D) or aversive zones (OF IZ, EPM OA, LDB-L) was found. The difference appeared to lie in which of the aversive compartments the mice would avoid. Mice sampled by cheek blood generally avoided the OF. Besides being a safe zone, the CA of the EPM tended to act as a hide, where the mouse could observe the other compartments of the test. Based on a natural aversion of open spaces, the entire OF may appear as an aversive zone seen from the EPM.

Mice sampled by tail blood generally avoided the LDB. However, the subdivision of time between the LDB-L and LDB-D did not differ significantly from the other groups. Generally, the mice seemed to just prefer the other compartments of the maze and explored the maze in a similar fashion to the control mice, as judged by the number of entries.

Interestingly, the mice sampled by ABS spent significantly less time in the CA of the EPM as compared to the control mice. Furthermore, although entering the EPM OA a comparable number of times to the mice of the other experimental groups, ABS mice would enter the EPM OA without prior risk assessment, which suggests less cautious locomotor activity.

Three components were extracted from the PCA: PC1 was, based on the factor loadings, interpreted as behaviors that seemed to explore the aversive zones of the test; time spent in the entire OF was positively correlated to time spent in the EPM OA as well as the total number of entries and negatively correlated to LDB-D. Therefore, PC1 was termed ‘exploration and curiosity’, which was expressed less by mice sampled by cheek blood than by mice sampled by tail blood. Confined to the EPM CA, we interpreted the behaviors of cheek-bled mice as more fearful compared with the other groups, which, in line with the above, indicates their general anxious behavior.

PC2 was, based on the positive correlations between entering the LDB-L, risk assessments in the EPM and the total number of entries with a negative correlation to risk assessments in the LDB, interpreted as ‘transit behavior after risk assessment’, and was expressed equally across groups, suggesting that this may be a reflex behavior in response to the risk assessment.

PC3 was interpreted as ‘anxiety’, based on a tendency in the factor loadings, where mice that would spend time in the EPM would not express much behavior elsewhere, which was negatively correlated to behaviors in the safe zone of the LDB (LDB-D). This is in line with the researchers’ observations that some mice would tend to stay in the CA of the EPM during the 15 min trial. Anxiety-like behavior was expressed less by the ABS mice than by the control mice, further indicating that ABS mice were less fearful in the test apparatus. This attenuated anxiety-like behavior expressed by the ABS mice may merely be a result of a longer study period due to the surgery and, hence, more handling. More studies are needed on this matter to be conclusive.

BW changes, plasma corticosterone, FCM and OF avoidance in a triple test indicate that facial vein phlebotomy results in a higher stress response than do ABS and caudal vein phlebotomy. Between the two manual blood sampling groups, the difference may be explained by the method of restraint rather than by the venipuncture itself. Mice subjected to facial vein phlebotomy were scruffed, punctured in the facial vein and then released after blood collection. This method was brief compared with caudal vein phlebotomy, but the latter method involved less restraint, with only the tail being handled during blood sampling. Thus, it is likely that the complete restraint of the animal required for blood sampling from the cheek resulted in higher stress levels as compared to tail blood sampling. Another possible explanation for the increased stress levels of mice subjected to facial vein phlebotomy may be related to pain, as the masseter muscle, which is the major masticatory muscle in rodents,^63^ is injured during blood sampling. As opposed to caudal vein phlebotomy, where a 25 gauge needle was used for the venipuncture, a Goldenrod lancet, which is five mm at the widest point, was used to puncture the facial vein. Puncturing of the facial vein may also be performed using a 25 gauge needle which, compared with a lancet, should cause a less wide trauma in the cheek. However, this has not yet been investigated and more studies on the tissue trauma after facial vein phlebotomy are needed.

Generally, cheek blood is an easy and quick method for obtaining blood, but controlling the bleeding could be difficult in some cases. With tail blood it was more difficult to obtain a volume of 50 µL, but in most cases enough blood was obtained and bleeding was easily controlled. This method is also considered easy, and a previous puncture in the tail could often be reused, saving the mice from multiple punctures of the tail veins. However, caudal vein phlebotomy is more time-consuming compared with cheek blood sampling. It should be pointed out that although caudal vein phlebotomy affected the stress response the least, mice in this group did show signs of acute stress, as evidenced by the increased concentration of plasma corticosterone compared with the control mice. Thus, even those blood sampling methods which have minimal effect on stress parameters will inevitably cause an acute stress response, which must be taken into consideration in all set-ups.

Mice sampled by ABS were significantly affected after surgery, on most parameters. However, after three days of recovery the mice did not show any signs of stress in relation to the blood samplings. An advantage with this method was that more samples could be obtained from each animal as compared to the manual sampling techniques, due to the precise control of the sample volume which the automated blood sampler allows. Although these mice had more samples drawn, the total blood loss of ABS mice (240 µL) approximated that of the mice sampled by cheek blood and tail blood (200–250 µL). Thus ABS may reduce the number of animals used in experiments which require a series of blood samples to be obtained, but this method requires surgical insertion of a catheter under anesthesia which is obviously a negative in terms of refinement in animal experimentation.^64^

Regardless of the sampling method, a skilled phlebotomist is required in order to minimize sampling-related trauma and unnecessary stress. The total volume sampled, the number of samples, and the frequency of sampling should be considered when planning a study.

In conclusion, repeated blood sampling of laboratory mice will inevitably affect physiological homeostasis through stress. This study demonstrates that routine blood sampling has a considerable impact on animal welfare. This must be taken into account when subjecting mice to repeated blood sampling. In addition, routine blood sampling techniques should be continuously refined, wherever they are applied. Based on the results obtained, facial vein phlebotomy seems less suitable for repeated blood sampling, as judged by physiological parameters as well as behavior. The choice between ABS and caudal vein phlebotomy should be based on study requirements, the skills of the researcher and the laboratory’s resources.
